# Machine learning combined with population pharmacokinetics: a hybrid model for predicting the plasma concentration of linezolid in critically ill pediatric patients

**DOI:** 10.3389/fphar.2026.1817282

**Published:** 2026-06-02

**Authors:** Yanping Zhang, Lin Zhu, Li Shen, Guangfei Wang, Junqi Zhang, Yang Chen, Yi Wang, Zhiping Li

**Affiliations:** 1 Department of Clinical Pharmacy, Children’s Hospital of Fudan University, National Children’s Medical Center, Shanghai, China; 2 Department of Neurology, Children’s Hospital of Fudan University, National Children’s Medical Center, Shanghai, China

**Keywords:** critically ill, linezolid, machine learning, pediatric patients, population pharmacokinetics, shap

## Abstract

**Objective:**

Linezolid is a crucial agent for treating drug-resistant Gram-positive bacterial infections in critically ill pediatric patients. However, its pharmacokinetics exhibit high inter-individual variability, making standard dosing regimens susceptible to underexposure or overexposure. This study aimed to explore the integration of population pharmacokinetics (PopPK) and machine learning (ML) algorithms to build a best-performing model for predicting individual linezolid plasma concentrations in critically ill children, thereby guiding personalized dosing.

**Methods:**

Based on data from a retrospective cohort of 145 critically ill pediatric patients (213 samples), 32 features (including PK parameters) and the target variable (linezolid plasma concentration) were included. A PopPK model was established, and Monte Carlo simulations were conducted to optimize dosing regimens. After systematically evaluating 7 ML algorithms, the best predictive model was identified. Its interpretation was then performed with SHapley Additive exPlanations (SHAP).

**Results:**

A one-compartment model with first-order elimination adequately described the pharmacokinetics of linezolid. Monte Carlo simulations for minimum inhibitory concentration (MIC) values between 0.5 and 2 mg/L, most renal function levels achieved a probability of target attainment (PTA) > 90% through dose adjustments. When MIC reached 4 mg/L, a PTA > 90% was achieved only in patients with severe renal impairment using 20 mg/kg q8h, yet with high safety risk, indicating the need for alternative antimicrobial agents. The LightGBM algorithm was best-performing among the seven algorithms evaluated, with testing set R^2^ = 0.777 and external validation set R^2^ = 0.686. However, these R^2^ values were optimistic, biased estimates inflated by the EBE-derived CL and V, which have been estimated using all available concentrations per patient, rather than conventional predictive performance. SHAP analysis revealed that clearance, time after dose, daily dose, weight, and serum creatinine were the top five most important features.

**Conclusion:**

This exploratory study integrated PK parameters into ML models for predicting linezolid plasma concentrations in critically ill pediatric patients, providing preliminary insights for future research as a proof of concept. However, the reported metrics could not support clinical deployment without leakage-free re-estimation using time-constrained or sequential validation.

## 1 Introduction

Linezolid, the first oxazolidinone antimicrobial agent (Diekema and Jones, 2001), is approved to treat severe infections in children (Jungbluth et al., 2003). It serves as a key therapeutic option for infections caused by multidrug-resistant Gram-positive bacteria, such as methicillin-resistant *Staphylococcus aureus* (MRSA) and vancomycin-resistant *Enterococcus* (VRE) (Diekema and Jones, 2001; Heidari and Khalili, 2023). Despite its increasing clinical application, linezolid exhibits significant inter-individual variability in its pharmacokinetic behavior in children, especially critically ill pediatric patients (Bandín-Vilar et al., 2022; Yang et al., 2021). This variability primarily stems from the complex pathophysiology of critically ill patients (Morales Castro et al., 2023; Dyer et al., 2024; Schlapbach et al., 2024) and the dynamic physiological development in children (Lyseng-Williamson and Goa, 2003), both of which can significantly affect the pharmacokinetics of linezolid. Consequently, traditional empirical dosing regimens based on weight or age often fail to achieve optimal pharmacodynamic targets (Yang et al., 2021). Linezolid has a narrow therapeutic window, with target trough concentrations (C_min_) of 2–7 mg/L for safety, while the efficacy target is defined as an area under the concentration-time curve over 24 h divided by the minimum inhibitory concentration (AUC_0-24_ h/MIC) ratio of ≥ 80. Insufficient exposure may lead to treatment failure, whereas excessive exposure is closely associated with myelosuppressive toxicity such as thrombocytopenia. (Abdul-Aziz et al., 2020).

Currently, therapeutic drug monitoring (TDM) for linezolid in patients is widely recommended, serving as a crucial tool for optimizing individualized dosing to balance efficacy and safety (Lin et al., 2022). However, TDM has inherent limitations due to its lag time, making it difficult to guide initial treatment and enable rapid dose adjustment (Minichmayr et al., 2024). Therefore, developing a model that can accurately predict the concentration of linezolid in real time is of great significance for optimizing medication guidance in critically ill children. Population Pharmacokinetic (PopPK) models are built upon a compartmental structure framework and incorporate both fixed and random effects to characterize the processes of drug absorption, distribution, metabolism, and excretion (ADME). The primary objective of PopPK models is to understand and quantify pharmacokinetic processes and their variability, as well as to identify covariates that explain such variability (Aljutayli et al., 2022; Keutzer et al., 2022).

Machine learning (ML), serving as a vital branch of artificial intelligence (AI), has shown great potential in supporting pharmacokinetic and pharmacodynamic analyses to guide individualized drug dosing (Ryan et al., 2024). Its ability to identify patterns and establish predictive models from complex, high-dimensional clinical data has achieved success in exposure prediction and dose optimization for various drugs (Woillard et al., 2021; Tang et al., 2024). However, the “black-box” nature of purely ML-based approaches also limits their pharmacological interpretability and clinical acceptability (Keutzer et al., 2022). To enhance interpretability in this context, recent studies have integrated PopPK parameters into ML models and applied SHapley Additive exPlanations (SHAP) analysis to trace predictions back to physiologically meaningful features such as clearance and volume of distribution, thereby enhancing interpretability while maintaining predictive accuracy (Chen et al., 2025; Liu et al., 2025).

Studies have explored the integration of PopPK with ML to achieve individualized dosing of voriconazole (Shen et al., 2025) and tacrolimus (Liang et al., 2025) in children. However, such a hybrid model has not yet been applied to predict linezolid concentration in critically ill pediatric patients. Therefore, the aim of this study was to build a linezolid PopPK model for critically ill children. Based on the PopPK model, individual CL and V will be extracted as features and combined with multiple ML algorithms to construct a prediction model of concentration. The predictive performance of different algorithms was systematically compared to explore potential individualized dosing strategies, providing preliminary methodologies and exploratory evidence for future research on linezolid therapy optimization in critically ill pediatric patients.

## 2 Materials and methods

### 2.1 Patients and data

A retrospective study was conducted on critically ill pediatric patients admitted to the Children’s Hospital of Fudan University from January 2022 to October 2025. Patients were included in this study according to the following criteria: 1) hospitalization in the pediatric intensive care units (PICU) and treatment with linezolid; 2) age < 18 years; 3) receipt of intravenous linezolid therapy for more than 3 days; 4) at least one TDM measurement was performed. Exclusion criteria were: 1) linezolid plasma concentration below the limit of quantification (BLOQ); 2) patient information was unavailable. The dosage of linezolid was 10 mg/kg every 8 or 12 h for patients under 12 years of age, and 600 mg every 12 h for patients aged 12 years and older, administered via intravenous infusion over 1–2 h. Routine TDM of linezolid is performed only at steady state. Blood samples of each patient at steady state were collected half an hour after the end of the infusion (peak concentration) and half an hour before the next scheduled administration (trough concentration). This study was approved by the Ethics Committee of the Children’s Hospital of Fudan University [No. (2025)506)] (Shanghai, China).

Linezolid concentration data were obtained through TDM records. Demographic characteristics and clinical information of the patients were collected from the hospital’s electronic medical record system, including gender, age, weight, height, body mass index (BMI), platelet count (PLT), white blood cell count (WBC), neutrophil percentage (NEUT%), red blood cell count (RBC), hemoglobin (HGB), total protein (TP), albumin (ALB), alanine aminotransferase (ALT), aspartate aminotransferase (AST), total bilirubin (TBIL), direct bilirubin (DBIL), serum creatinine (SCR), international normalized ratio (INR), fibrinogen (FIB), prothrombin time (PT), activated partial thromboplastin time (APTT), and C-reactive protein (CRP). Additionally, whether patients received extracorporeal membrane oxygenation (ECMO) and continuous renal replacement therapy (CRRT) support, as well as concomitant medications including meropenem (MEM), omeprazole (OME), fluconazole (FLU), and voriconazole (VRC) were also recorded in detail. The estimated glomerular filtration rate (eGFR) was determined using the Schwartz formula (Schwartz et al., 1984, Schwartz et al., 2009) (Equation 1). The k is 0.45 for children under 1 year of age and 0.413 for children aged 1 year or older.

$$\text{eGFR }\left({mL/\min/1.73\;m}^{2}\right)=\frac{k\;*\;H\text{eight}\;\left(\text{cm}\right)}{S\text{cr}\;\left(\text{mg}/\text{dL}\right)}$$ (1)

### 2.2 Therapeutic drug monitoring of linezolid

Linezolid concentrations were determined using a validated high-performance liquid chromatography (HPLC) method (LC-2050C 3D; Shimadzu Inc.) with ultraviolet detection. The chromatographic column was a Waters XBridge C18 (4.6 × 150 mm, 5 μm). The detection wavelength was set at 253 nm. The mobile phase consisted of water (phase A) and methanol (phase B) at a volume ratio of 64:36. The flow rate was maintained at 1.0 mL/min. A linear calibration curve was established for concentrations spanning from 0.25 mg/L to 50.00 mg/L, and the lower limit of quantification was 0.25 mg/L.

### 2.3 Data collection and processing

During the data cleaning phase, missing values in continuous variables (each with a missing rate below 10%) were handled using median imputation, while categorical variables had no missing values and were appropriately encoded, resulting in a complete dataset of 213 × 33 (213 rows representing TDM samples, and 33 columns comprising 32 features and 1 target variable). We designated linezolid plasma concentrations as the target variable and randomly partitioned the dataset into training and testing sets using a 7:3 split with a random seed of 40 to ensure reproducibility. Additionally, an external validation dataset was collected, which included 13 TDM measurements from 10 patients enrolled between November 2025 and December 2025, to evaluate the predictive performance of the optimal model.

### 2.4 Population pharmacokinetic modeling

A PopPK analysis of linezolid was conducted using the NONMEM software (Version VII, Icon Development Solutions, United States of America). Parameter estimation was performed using the first-order conditional estimation method with interaction (FOCE-I), and a one-compartment model with first-order elimination was employed to characterize the pharmacokinetics of linezolid. Inter-individual variability was described using an exponential error model, while residual variability was modeled using a proportional error structure.

Covariate selection was carried out through a stepwise approach involving forward inclusion and backward elimination. During forward inclusion, a covariate was incorporated into the model if it reduced the objective function value (OFV) by more than 3.84 (p < 0.05, df = 1). In the backward elimination phase, a covariate was retained in the final model if its removal increased the OFV by more than 10.83 (p < 0.001, df = 1).

The final model was validated and evaluated using goodness-of-fit diagnostic plots, bootstrap method, prediction- and variability-corrected visual predictive check (pvcVPC), and normalized prediction distribution errors (NPDE) test. Additionally, we compared our newly developed PopPK model with a previously published linezolid PopPK model for critically ill pediatric patients (Yang et al., 2021) in terms of model structure and predictive performance. The predictive performance was evaluated using the following metrics: relative prediction error (PE%), median prediction error (MDPE), median absolute prediction error (MAPE), and the percentage of PE% within ±20% (F_20_) and ±30% (F_30_).

Based on the established final model, individual pharmacokinetic parameters CL and V were calculated using the empirical Bayesian estimations (EBE) method via the POSTHOC option in NONMEM, utilizing all available data from each patient. These estimated parameters were subsequently incorporated as additional features in the following machine learning modeling process.

### 2.5 Simulation and optimization of dosing regimen

To optimize the dosing regimen, Monte Carlo simulations (n = 1000) were performed. We calculated the probability of target attainment (PTA) under different dosing regimens for critically ill pediatric patients with varying renal function, where the target was defined as achieving AUC_0–24 h_/MIC ≥ 80 (Abdul-Aziz et al., 2020). PTA ≥ 90% is considered the optimal choice for antimicrobial efficacy (Yang et al., 2021). In addition, a C_min_ of 7 mg/L was selected as the safety threshold (Abdul-Aziz et al., 2020). For each simulated scenario, the probability of exceeding this safety threshold was calculated. The simulated MIC values (0.5, 1, 2, and 4 mg/L) were selected based on the Clinical and Laboratory Standards Institute (CLSI) standards (Performance Standards for Antimicrobial Susceptibility Testing, 2026). The AUC_0–24 h_ was calculated from EBE.

### 2.6 Machine learning modeling and validation

Feature selection using an XGBoost regressor was performed to identify the most relevant predictors of linezolid plasma concentration. Feature importance was calculated and ranked based on the XGBoost algorithm. Then, features were added stepwise in order of importance, and the average coefficient of determination (R^2^) value was computed via cross-validation at each step. The feature combination that yielded the highest R^2^ was subsequently selected for final model training. To construct and screen for the best predictive model, 7 ML algorithms were employed, including extreme gradient boosting (XGBoost), light gradient boosting machine (LightGBM), random forest (RF), adaptive boosting (AdaBoost), support vector regression (SVR), gradient boosting decision tree (GBDT), and categorical boosting (CatBoost). Hyperparameter tuning was performed using grid search combined with 5-fold cross-validation (Alhakeem et al., 2022).

Four evaluation metrics were used to assess the fitting capability and predictive performance of the machine learning models: mean absolute error (MAE), mean squared error (MSE), root mean squared error (RMSE), and the coefficient of determination (R^2^) (Yao et al., 2025). The calculation formulas are provided in Equations 2–5. R^2^ was applied to evaluate how well the model fits the observations, with a value range from 0 to 1. The closer the value is to 1, the better the model’s fitting performance. Correspondingly, lower MAE, MSE, and RMSE values are associated with enhanced predictive performance. Based on these metrics, the optimal model was selected.

$$\text{MAE}=\frac{1}{n}\sum_{i=1}^{n}\left|y_{i}‐{\hat{y}}_{i}\right|$$ (2)

$$\text{MSE}=\frac{1}{n}\sum_{i=1}^{n}{\left(y_{i}‐{\hat{y}}_{i}\right)}^{2\;}$$ (3)

$$\text{RMSE}=\sqrt{\frac{1}{n}\sum_{i=1}^{n}{\left(y_{i}‐{\hat{y}}_{i}\right)}^{2}}$$ (4)

$$R^{2}=1‐\frac{\sum_{i=1}^{n}{\left(y_{i}‐{\hat{y}}_{i}\right)}^{2}}{\sum_{i=1}^{n}{\left(y_{i}‐\bar{y}\right)}^{2}}$$ (5)

$y_{i}$ is the observed concentration, ${\hat{y}}_{i}$ is the predicted concentration, $\bar{y}$ is the mean of observed concentrations.

**FIGURE 1:**
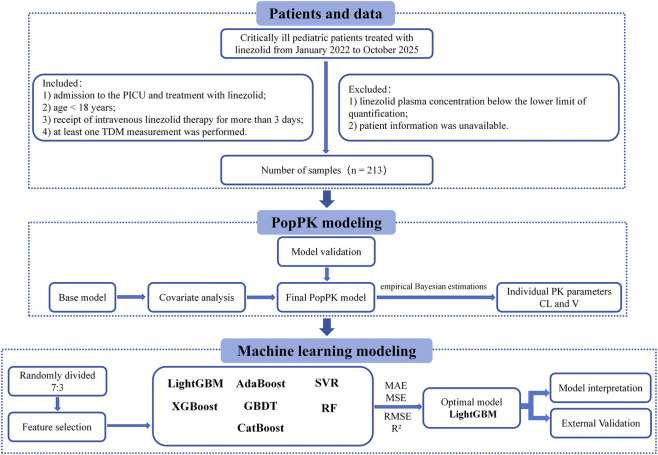
The workflow for developing a hybrid model.

Subsequently, to gain a deeper understanding of the model’s decision-making mechanism and enhance its interpretability, we applied the SHapley Additive exPlanations (SHAP) method. SHAP values, which are derived from cooperative game theory, quantify each feature’s contribution to the model’s predictions and provide both local and global interpretability. Compared with permutation importance, SHAP handles feature correlations more robustly and reveals the direction (positive or negative) of each feature’s effect. SHAP visualizations were generated to illustrate the contribution and influence patterns of features on the model predictions (Redlich et al., 2026; Zhang et al., 2025). Figure 1 shows the workflow for developing a hybrid model.

### 2.7 Statistical analysis

Continuous variables were presented as medians and interquartile ranges [Median (IQR)]. Non-normally distributed data were analyzed using the Mann-Whitney U test, while normally distributed data were analyzed using the independent samples t-test. Categorical variables were described as frequencies (n, %) and analyzed using the chi-square test. Statistical significance was defined as a two-sided p-value < 0.05. All analyses were performed using Python (version 3.13.5) and R software (version 4.5.1).

## 3 Results

### 3.1 Baseline information of pediatric patients

A total of 213 steady-state linezolid concentrations (39 peak and 174 trough concentrations) obtained from 145 critically ill pediatric patients were used for the development, training, and testing of the hybrid model. Among the patients, 69 (47.6%) were diagnosed with severe pneumonia, 43 (29.7%) with sepsis, 19 (13.1%) with central nervous system infection, and the remaining 14 (9.6%) with other conditions. Five BLOQ samples were excluded. According to the literature (Beal, 2001; Keizer et al., 2015), when the proportion of BLOQ samples is very small, the simple discard method (M1) performs as well as other more complex methods for handling BLOQ data. The baseline information for the 32 feature variables (including PK parameters CL and V) and the target variable (linezolid concentration) in both the training and testing sets is presented in Table 1.

**TABLE 1:** The description of baseline information.

Variables	Values		p value
	Training set (n = 149)	Testing set (n = 64)	
Linezolid concentration (mg/L)	5.75 (2.24–10.70)	5.23 (2.40–13.60)	0.76
Daily dose (mg)	600.00 (240.00–930.00)	600.00 (371.25–877.50)	0.52
TAD (h)	7.50 (7.50–7.50)	7.50 (7.50–7.50)	0.63
CL (L/h)	2.69 (1.27–3.95)	2.39 (1.55–3.66)	0.92
V (L)	15.87 (7.82–23.55)	15.23 (9.91–21.75)	0.71
Age (years)	6.60 (1.20–10.30)	6.20 (2.63–10.10)	0.74
Gender (n, %)	​	​	0.37
Male	86 (57.72)	32 (50.00)	​
Female	63 (42.28)	32 (50.00)	​
Weight (kg)	21.00 (9.10–33.50)	20.00 (12.03–30.50)	0.71
BMI (kg/m^2^)	15.72 (14.15–18.35)	15.52 (14.23–18.39)	0.86
SCR (μmol/L)	28.90 (20.80–42.90)	32.80 (23.45–45.48)	0.15
eGFR (mL/min/1.73 m^2^)	124.98 (90.43–171.96)	116.06 (84.02–153.46)	0.23
TBIL (μmol/L)	8.40 (4.80–18.90)	11.40 (7.28–19.35)	0.07
DBIL (μmol/L)	3.80 (2.10–8.80)	4.60 (2.50–9.68)	0.15
ALT (U/L)	21.80 (13.69–61.19)	22.65 (13.52–54.16)	0.98
AST (U/L)	39.76 (25.29–60.34)	38.92 (28.01–66.91)	0.57
ALB (g/L)	36.16 (32.9–39.73)	35.93 (33.21–39.13)	0.89
TP (g/L)	59.80 (53.30–66.86)	59.47 (54.80–67.01)	0.95
WBC (×10^9^/L)	8.26 (5.16–12.43)	7.78 (4.88–11.48)	0.48
NEUT%	69.90 (55–78.9)	70.60 (48.78–77.93)	0.42
RBC (×10^12^/L)	3.05 (2.57–3.47)	2.94 (2.62–3.35)	0.57
HGB (g/L)	85.00 (75.00–96.00)	84.00 (76.00–96.00)	0.54
PLT (×10^9^/L)	212.00 (78.00–346.00)	198.50 (103.75–345.00)	0.92
CRP (mg/L)	14.00 (3.45–42.45)	15.32 (3.91–56.89)	0.73
PT (s)	13.90 (13.10–15.10)	14.25 (13.00–15.73)	0.43
APTT (s)	40.00 (33.90–46.20)	39.10 (35.98–43.30)	0.59
FIB (g/L)	2.73 (1.81–3.90)	2.80 (1.82–3.83)	0.91
INR	1.05 (0.98–1.19)	1.09 (0.98–1.25)	0.42
Concomitant medications (n, %)			
Meropenem	77 (51.68)	37 (57.81)	0.50
Omeprazole	60 (40.27)	28 (43.75)	0.75
Voriconazole	21 (14.09)	8 (12.50)	0.93
Fluconazole	29 (19.46)	12 (18.75)	0.55
Extracorporeal life support (n, %)			
CRRT	17 (11.41)	3 (4.69)	0.71
ECMO	9 (6.04)	3 (4.69)	0.20

Data are presented as median (IQR) for continuous variables and as n (%) for categorical variables. Trough samples at 7.5 h dominate the datasets (approximately 68% of the training set and 75% of the testing set), causing the median and quartiles to all equal 7.5 h despite the presence of other TAD values.

### 3.2 Population pharmacokinetic analysis

The analysis showed that the pharmacokinetics of linezolid were best characterized by a one-compartment model with first-order elimination. A proportional model was used to explain the residual variability. The covariate analysis identified that weight and eGFR were significant covariates for clearance (CL). Weight was the significant covariate for volume of distribution (V). The following equations (Equations 6 and 7) represent the final PopPK model:

$$\text{CL}\left(L/h\right)=2.80\times {\left(\text{WT}/20.00\right)}^{0.69}\times {\left(\text{eGFR}/126.39\right)}^{0.34}$$ (6)

$$V\left(L\right)=14.48\times {\left(\text{WT}/20.00\right)}^{0.89}$$ (7)

The robustness of the final PopPK model was verified using a nonparametric bootstrap (n = 1000). Minimization success rate was 99.9%. The median and 95% CI of the parameters estimated from the bootstrap analysis in Table 2 aligned well with the final model. The goodness-of-fit plots are shown in Supplementary Figure S1. Supplementary Figure S2 presents the pvcVPC results. The majority of observations fell within the 95% prediction intervals of the simulated data, suggesting a robust predictive ability of the final PopPK model. The results of NPDEs test are shown in Supplementary Figure S3. The NPDEs test exhibited a distribution and density pattern conforming to normality, suggesting the final model adequately described the individual data. This was supported by statistical tests: the P values for the t-test, Shapiro-Wilk test, Fisher’s variance test, and the global normality test were 0.285, 0.408, 0.208, and 0.623, respectively.

**TABLE 2:** Parameter estimates of linezolid and bootstrap validation.

Parameter	Final model		Shrinkage (%)	Bootstrap		Bias (%)
	Estimation	RSE (%)		Median	95%CI	
CL (L/h) = θ_1_ × (WT/20.00) ^θ3^ × (eGFR/126.39) ^θ4^						
θ_1_	2.80	8.40	​	2.74	(2.32, 3.27)	−2.14
θ_3_	0.69	12.60	​	0.69	(0.48, 0.90)	0
θ_4_	0.34	23.10	​	0.34	(0.20, 0.54)	0
V (L) = θ_2_ × (WT/20.00) ^θ5^						
θ_2_	14.48	17.60	​	14.08	(9.73, 20.30)	−2.76
θ_5_	0.89	17.50	​	0.89	(0.43, 1.30)	0
Inter-individual variability (%)						
ω_CL_ (%)	48.79	11.20	6	47.43	(37.28, 57.96)	−2.79
Residual variability						
σ_PROP_ (%)	40.12	9.40	24	39.37	(31.46, 47.75)	−1.87

Abbreviations: V, volume of distribution; CL, clearance; ω_CL_, inter-individual variability of CL; σ_PROP_, proportional residual variability; RSE, relative standard error; Bias, prediction error, Bias=(Median-Estimate)/Estimate×100%; 95% confidential interval is displayed as the 2.5, 97.5 percentile of bootstrap estimates.

The comparison results of the two PopPK models are presented in Supplementary Table S3 and Supplementary Figure S5. The results showed that our PopPK model demonstrated better fit to our sparse trough-dominant dataset than the two-compartment model of Yang et al., which is over-parameterised for this sampling design. These findings confirm the need for developing a new PopPK model for our patient population.

### 3.3 Simulation and dosing regimen optimization

**FIGURE 2:**
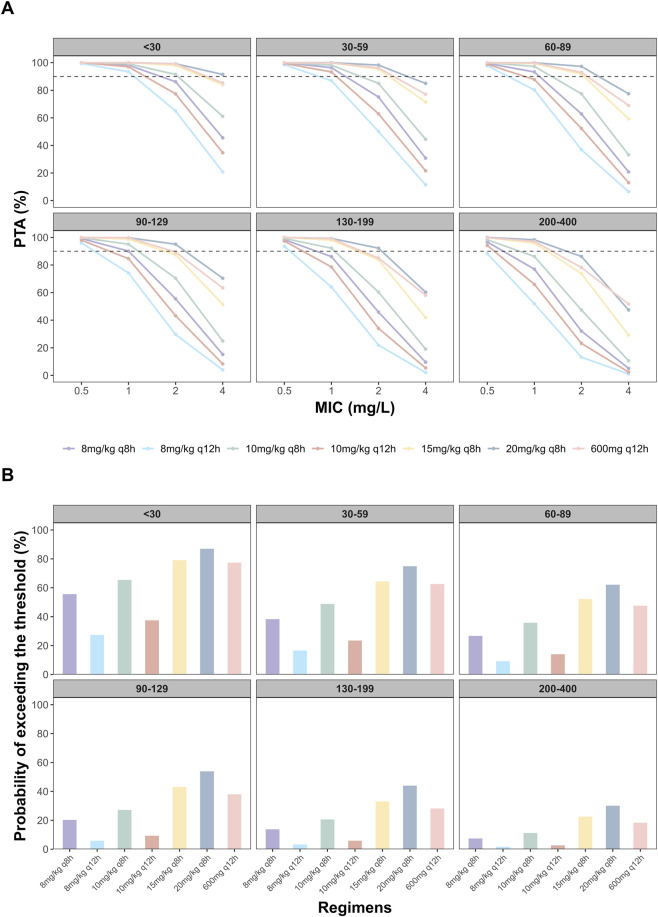
The results of Monte Carlo simulations. **(A)** PTA for different dosing regimens across varying levels of renal function and MIC values. **(B)** Probability of exceeding the safety threshold (C_min_ = 7 mg/L) for different dosing regimens across varying levels of renal function and MIC values. The levels of renal function are eGFR<30,30–59,60–89,90–129,130–199,200–400 mL/min/1.73 m^2^.

Figure 2A depicts the association between dosing regimens and the PTA across varying levels of renal function and MIC values. Figure 2B displays the probability of exceeding the safety threshold (C_min_ = 7 mg/L) across various dosing regimens. The dosing regimen was selected by comprehensively balancing efficacy and safety, as higher doses increased the PTA but also significantly raised the risk of exceeding the safety threshold. When the MIC was 0.5 mg/L, a regimen of 8 mg/kg q12h achieved a PTA of nearly 90% in most cases. For an MIC of 1 mg/L, the recommended regimens were 8 mg/kg q12h, 10 mg/kg q12h, 8 mg/kg q8h, 10 mg/kg q8h, and 15 mg/kg q8h for eGFR levels of <30, 30–59, 60–89, 90–199, and 200–400 mL/min/1.73 m^2^, respectively. At an MIC of 2 mg/L, regimens of 15 mg/kg q8h or 600 mg q12h achieved a PTA above 90% when eGFR was 30–89 mL/min/1.73 m^2^, while a regimen of 20 mg/kg q8h was required to reach a PTA > 90% when eGFR exceeded 90 mL/min/1.73 m^2^. For an MIC of 4 mg/L, only patients with eGFR < 30 mL/min/1.73 m^2^ attained a PTA above 90% (91.5%) with 20 mg/kg q8h, while the probability of exceeding the safety threshold was as high as 87%. Therefore, alternative antimicrobial agents were considered.

### 3.4 Machine learning modeling and validation

The best feature subset was determined via rigorous feature selection, as shown in Supplementary Figure S4. Twelve features, including CL, PLT, Daily Dose, SCR, WT, time after dose (TAD), FIB, RBC, WBC, ALT, TP, and HGB, were finally selected as important variables for training different ML models.

**FIGURE 3:**
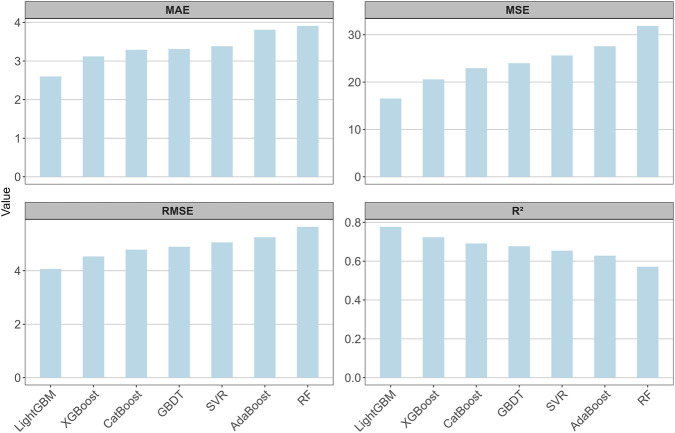
Comparison of predictive performance of different ML algorithms on the testing set. The range for R^2^ is between 0 and 1, where a higher R^2^ and lower values of MAE, RMSE, and MSE indicate better model performance.

**FIGURE 4:**
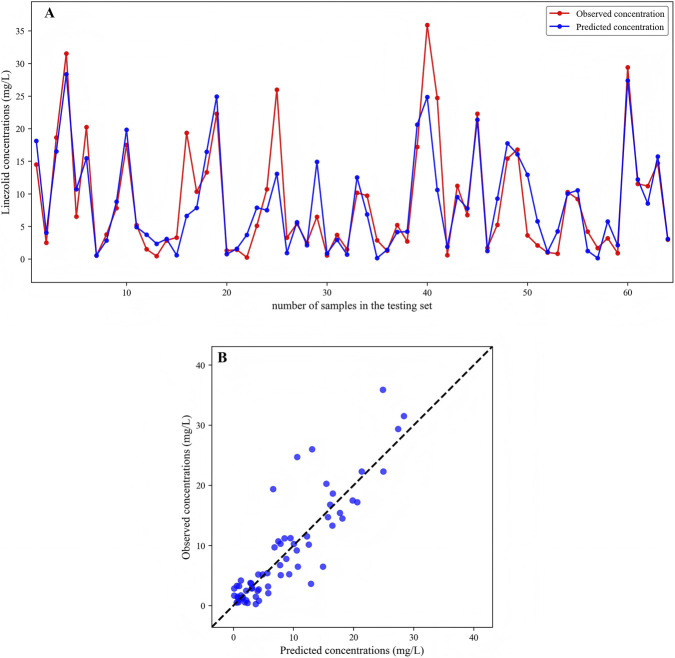
Predicted versus observed linezolid concentrations in the testing group. **(A)** The predicted concentrations are indicated by the blue dots, and the observed concentrations are indicated by the red dots. The X-axis corresponds to the number of samples in the testing set. **(B)** The scatter plots of predicted versus observed linezolid concentrations. The predicted concentrations are individual predictions, as they are generated using individual CL derived from the EBE.

The predictive performance metrics of the different ML models on the testing set are presented in Supplementary Table S1 and Figure 3. The best-tuned hyperparameters for ML models are shown in Supplementary Table S4. Among the seven evaluated algorithms, LightGBM was identified as the best model. It achieved the highest R^2^ of 0.777, with the lowest MAE, MSE, and RMSE of 2.599, 16.532, and 4.066, respectively, indicating a high goodness of fit. Figures 4A,B display the distributions of predicted versus observed linezolid concentrations for each patient in the testing set using the LightGBM model. The external validation results (R^2^ = 0.686, MAE = 5.240, MSE = 37.847, and RMSE = 6.152) offered preliminary and exploratory evidence for the LightGBM model’s performance. The demographic characteristics and clinical information of the patients in the external validation dataset are presented in Supplementary Table S2.

**FIGURE 5:**
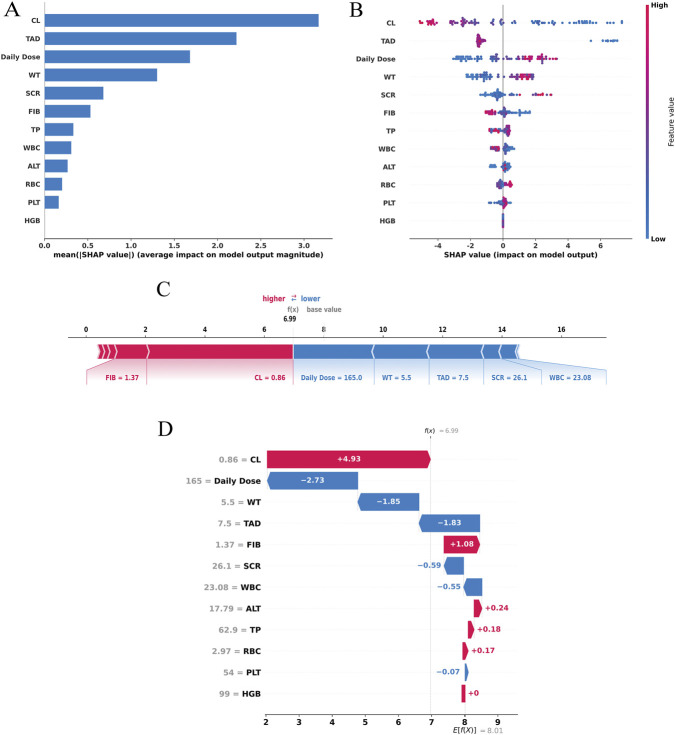
The SHapley Additive exPlanations (SHAP) analysis of the LightGBM. **(A)** Bar plot of the twelve variables according to the mean (|SHAP value|). **(B)** The beeswarm plot shows the SHAP values for twelve features. **(C)** The force plot shows the directional impact of features on an individual prediction. **(D)** The waterfall plot quantifies the stepwise contribution of each feature to an individual prediction.

**FIGURE 6:**
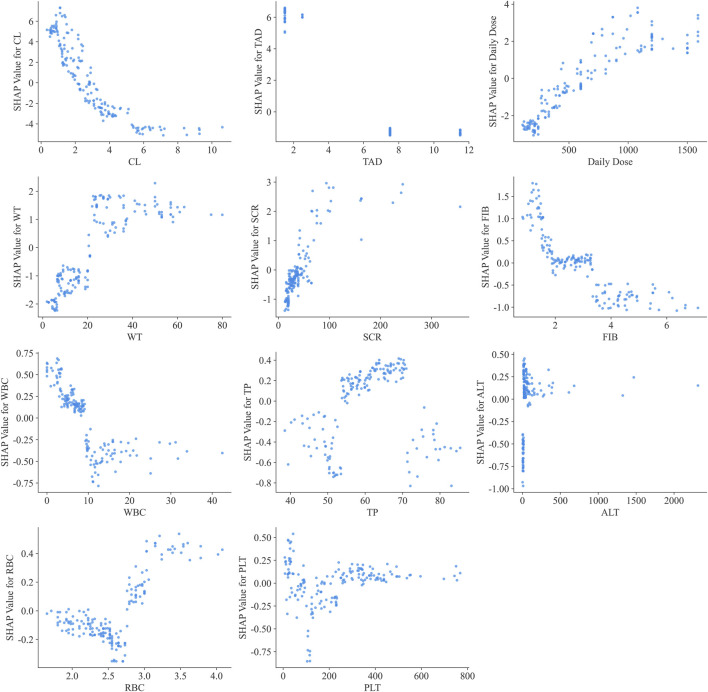
The SHAP dependence plots for the LightGBM model. Illustrating the impact of key features on model output, where positive SHAP values for specific features indicate an increase in linezolid concentration.

Interpretability analysis was conducted on the LightGBM model. SHAP plots illustrated the contribution of each feature to predictions of the model, with features ranked by their importance. The mean absolute SHAP values for the key features are shown in Figure 5A. They were ranked by importance as follows: CL, TAD, Daily Dose, WT, SCR, FIB, and others. The beeswarm plot in Figure 5B shows how each feature influences the final predictions, in terms of both the strength and direction of its effect. The color of the points represented the specific value of each feature. The red points indicated higher values and the blue points indicated lower values. The horizontal axis plotted each feature value against its individual SHAP value. Negative and positive influences were positioned to the left and right of the origin, respectively, while the central area indicated a weak or negligible effect. The wide distribution of SHAP values for CL, TAD, Daily Dose, and WT indicated that these features have a significant impact on the predictions. Figure 5C (force plot) and **5D** (waterfall plot) provide SHAP interpretations for an individual sample. Starting from a baseline prediction value E [f(x)] = 8.01 mg/L, the output was progressively adjusted by the contributions of each feature, resulting in a final model output of f(x) = 6.99 mg/L. The SHAP dependence plots (Figure 6) identified lower CL and TAD, and higher Daily Dose, WT, SCR, and ALT as factors associated with elevated linezolid concentrations.

### 3.5 Conceptual framework for potential clinical application

**FIGURE 7:**
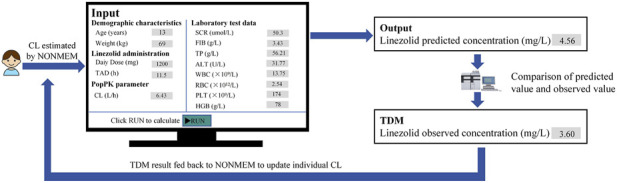
Closed-feedback workflow for predicting linezolid concentrations using the LightGBM model.

Figure 7 shows a conceptual workflow of predicting the concentration of linezolid in individual critically ill children using the LightGBM model. In this proposed framework, the PK parameter CL would be derived using NONMEM via EBE, then merged with other features to predict concentration. The prediction would be compared with subsequent TDM measurements, and the prediction error would be fed back into the Bayesian framework to update the PK parameters. This iterative process would theoretically enable continuous model refinement. However, we emphasize that the current study is exploratory and does not validate this framework for real-time clinical use.

## 4 Discussion

This study explored the integration of PopPK with ML algorithms to construct a hybrid model for predicting concentrations and optimizing linezolid dosing strategies in critically ill children with severe infections in the PICU. First, we built a robust PopPK model based on real-world data to quantify the influence of key covariates and conducted dosing regimen simulations based on renal function. Second, we incorporated the individualized pharmacokinetic parameters (CL and V) derived from the PopPK model as features into ML models, ultimately selecting LightGBM as the best-performing predictive model. This integrated framework achieved a high R^2^ of 0.777 and the model’s results were made clinically interpretable through SHAP analysis. Our research confirmed that for critically ill children, ML models integrated with pharmacological knowledge may reliably guide the individualized dosing of linezolid, representing a step toward precision anti-infection treatment.

The linezolid PopPK model established was best described by a one-compartment model with first-order elimination in this study. This was different from the model established by Yang et al. (2021) for linezolid in critically ill children, which employed a two-compartment model developed from denser sampling (including intermediate time points). Our dataset, derived from routine TDM, contained predominantly trough concentrations with limited distribution-phase information, necessitating the simpler one-compartment structure. Moreover, when the two-compartment model by Yang et al. (2021) was externally validated on our sparse dataset, it did not fit our data well. This is likely because the two-compartment structure is over-parameterized for sparse data, leading to unstable estimates. These findings support the development of a new PopPK model tailored to our specific data characteristics.

Weight and eGFR were identified as key covariates affecting CL, which corresponded to the findings of Li et al. (2019) and Tian et al. (2025) Children are in a stage of growth and development, where body size is a primary determinant of CL in children (Back et al., 2019). Furthermore, approximately 30% of linezolid is excreted through the kidneys in its original form (Zavřelová et al., 2025). A growing number of studies indicate that linezolid dosing should be adjusted based on renal function to reduce the occurrence of hematotoxicity (Jones et al., 2015; Liu et al., 2022). In the PICU, a wide spectrum of renal function is commonly observed in children, ranging from markedly reduced clearance due to acute kidney injury (AKI) (Kaddourah et al., 2017) to augmented renal clearance (ARC) driven by elevated cardiac output resulting from fluid resuscitation and vasopressor support (Rhoney et al., 2021). Based on this, we conducted simulations of various linezolid dosing regimens under different renal function and MIC values using our final established PopPK model. For MIC values ranging from 0.5 to 2 mg/L, most renal function levels achieved a PTA > 90% through dose adjustments (e.g., 8–15 mg/kg q8h or q12h). When the MIC increased to 4 mg/L, even a dosage of 20 mg/kg q8h could not achieve a PTA of 90% for patients with eGFR > 30 mL/min/1.73 m^2^, which was consistent with the findings reported by Li et al. (2019) However, increasing the dose also raised the probability of exceeding the safety threshold (C_min_ = 7 mg/L), potentially leading to the occurrence of thrombocytopenia toxicity. Therefore, adjusting the dosing regimen should involve a comprehensive balance between efficacy and safety.

The PopPK model is based on physio-pharmacological principles, with parameters having clear biological significance, and is adept at parsing sources of variability from sparse data and deducing population patterns. However, its accuracy in predicting individual concentration may be limited by the relatively fixed model structure and uncertainty of random effect estimation (Mould and Upton, 2012). On the other hand, ML algorithms demonstrate strong predictive capabilities due to their nonlinear fitting abilities and the advantage of automatically learning complex patterns from high-dimensional data. However, their “black-box” nature remains a concern when applying them to medical decision-making (Sidey-Gibbons and Sidey-Gibbons, 2019). Our strategy integrated the strengths of both approaches. Among the 7 ML algorithms compared, LightGBM emerged as the best-performing model. LightGBM is the improved version of the gradient boosting framework. While preserving the high prediction accuracy of gradient boosting, it adopts a leaf wise growth strategy and introduces two key techniques: gradient based one side sampling and exclusive feature bundling. These accelerate training speed and reduce resource consumption (Zhang et al., 2019). Its predictive performance indicated that the model may capture the complex nonlinear relationships influencing linezolid plasma concentrations in critically ill children.

The clinical value of a model lies not only in its predictive accuracy but also in the transparency and interpretability of its decision-making process. SHAP analysis was applied to interpret the LightGBM model, which clearly and quantitatively revealed the direction and contribution of each feature to the final predicted concentration. SHAP analysis results showed that CL was the most important feature for predicting plasma concentration, with its SHAP value was higher than that of other variables. A higher CL directly predicted a lower plasma concentration, which was consistent with other studies and aligned with the fundamental principles of pharmacokinetics (Ma et al., 2024; Liu et al., 2025). Administration information, including daily dose and TAD, was found to be highly significant because this information is directly related to the plasma concentration of linezolid. TAD enabled the predicted value to reflect the plasma level at the corresponding time of dose administration and blood sampling. Weight plays a crucial role in individualized linezolid dosing strategies for pediatric populations. Linezolid pharmacokinetics are age-dependent, with dosing for children under 12 years primarily based on weight. Compared to adults, children under 12 years exhibit a smaller area under the concentration-time curve and faster clearance (Lyseng-Williamson and Goa, 2003). Weight serves as a covariate representing body size and is well-established to significantly influence drug disposition (Guk et al., 2017). Studies have shown that both liver and kidney function affect the clearance of linezolid (Jones et al., 2015; Liao et al., 2023). Higher values of ALT and SCR, which served as indicators of liver and kidney function, were associated with higher predicted concentrations of linezolid. In contrast, higher values of FIB and WBC were associated with lower predicted concentrations of linezolid. This could be attributed to the close association between systemic inflammatory response syndrome and ARC (Sime et al., 2015), which enhanced drug clearance. This finding indicated that systemic inflammatory status also served as an important hidden covariate that could not be overlooked in the disposition of linezolid in critically ill children.

Several limitations of this study must be acknowledged. First, the retrospective, single-center design and limited sample size limit the generalizability of our findings, given the high heterogeneity among critically ill pediatric patients. In particular, the external validation set consisted of only 10 patients, which reduced the precision of our performance estimates. Furthermore, the case mix of our cohort (predominantly severe pneumonia and sepsis) may not reflect that of other PICUs in different regions. Therefore, future studies with larger and multi-center validation cohorts are necessary to rigorously evaluate the clinical utility of our model before widespread application. Second, in terms of sampling design, the data for this retrospective study were derived from routine TDM, resulting in sparse and predominantly trough concentration sampling points. While this approach may support the estimation of CL, it fails to fully capture the distribution kinetics of the drug in the body, leading to significant uncertainty in the estimation of key parameters such as V. Third, this study incorporated ECMO and CRRT only as binary covariates (yes/no) in the model, without integrating specific treatment parameters (e.g., flow rates and oxygenator types for ECMO, replacement fluid rates, and operational modes for CRRT). These parameters are dynamic and exert a substantial influence on drug CL and V (Ide et al., 2018; Milaković et al., 2024). This simplified approach may significantly underestimate the pharmacokinetic variability among patients under these treatment modalities. Fourth, a limitation concerning potential data leakage must be acknowledged. Individual CL and V were estimated using all available concentration data from each patient via the EBE method. When these PK parameters were used to predict concentrations at earlier time points, information from later time points was indirectly incorporated, which constitutes data leakage and leads to a certain degree of overfitting. This data leakage issue also affected the external validation metrics, as the same EBE workflow was applied to the external dataset. Future studies should adopt time-constrained validation schemes using only historical data to estimate PK parameters for sequential predictions, thereby avoiding data leakage.

## 5 Conclusion

In this exploratory study, PopPK was combined with ML to build a predictive model for linezolid plasma concentrations in critically ill pediatric patients. A one-compartment model with first-order elimination was developed, showing that WT and eGFR significantly influenced CL. Linezolid dosing should be adjusted based on MIC values and renal function. The LightGBM algorithm was best-performing among the seven algorithms evaluated, with testing set R^2^ = 0.777 and external validation set R^2^ = 0.686. However, these R^2^ values were optimistic, biased estimates inflated by the EBE-derived CL and V, which have been estimated using all available concentrations per patient, rather than conventional predictive performance. SHAP analysis revealed that CL, TAD, Daily Dose, WT, and SCR were significant predictors in the model. This framework served only as a proof of concept, and while the preliminary findings provided a reference for future research, the reported metrics could not support clinical deployment without leakage-free re-estimation using time-constrained or sequential validation.

## Data Availability

The original contributions presented in the study are included in the article/Supplementary Material, further inquiries can be directed to the corresponding authors.
