# Supplementary material for: Machine learning combined with population pharmacokinetics: a hybrid model for predicting the plasma concentration of linezolid in critically ill pediatric patients
Source: Front Pharmacol. 2026 Jun 2;17:1817282. doi: 10.3389/fphar.2026.1817282 (PMC13269215; doi:10.3389/fphar.2026.1817282)
Supplement: Supplementary file 1 [file DataSheet1.docx]

**Supplementary Table S1** The comparison of performance of different ML algorithms on the testing set

| **Model** | **MAE** | **MSE** | **RMSE** | **R^2^** |
| --- | --- | --- | --- | --- |
| LightGBM | 2.599 | 16.532 | 4.066 | 0.777 |
| XGBoost | 3.121 | 20.557 | 4.534 | 0.723 |
| RF | 3.909 | 31.855 | 5.644 | 0.571 |
| SVR | 3.384 | 25.624 | 5.062 | 0.654 |
| AdaBoost | 3.813 | 27.583 | 5.252 | 0.628 |
| GBDT | 3.312 | 23.981 | 4.897 | 0.677 |
| CatBoost | 3.292 | 22.925 | 4.788 | 0.691 |

**Supplementary Table S2** Demographic characteristics and clinical information of patients in the external validation dataset

| **Variables** | **Median or Number** | **IQR** |
| --- | --- | --- |
| Patients/Samples | 10/13 |  |
| Linezolid concentration (mg/L) | 3.60 | 1.98-15.65 |
| Daily dose (mg) | 622.50 | 318.75-1275.00 |
| TAD (h) | 7.50 | 4.50-7.50 |
| Age (years) | 4.00 | 1.10-9.00 |
| Gender |  |  |
| Male | 5 |  |
| Female | 5 |  |
| Weight (kg) | 19.50 | 9.25-50.00 |
| BMI (kg/m^2^) | 17.42 | 14.98-21.93 |
| SCR (μmol/L) | 38.30 | 31.65-49.65 |
| eGFR (mL/min/1.73 m²) | 79.12 | 58.46-168.68 |
| TBIL (μmol/L) | 22.70 | 12.35-104.60 |
| DBIL (μmol/L) | 22.20 | 6.55-58.10 |
| ALT (U/L) | 31.77 | 20.79-90.50 |
| AST (U/L) | 65.49 | 34.56-167.54 |
| ALB (g/L) | 37.38 | 33.50-40.50 |
| TP (g/L) | 56.21 | 46.80-61.40 |
| WBC (×10^9^/L) | 7.91 | 6.17-8.74 |
| NEUT% | 65.40 | 52.45-77.45 |
| RBC (×10^12^/L) | 3.30 | 2.59-3.78 |
| HGB (g/L) | 95.00 | 80.00-116.00 |
| PLT (×10^9^/L) | 163.00 | 66.00-256.00 |
| CRP (mg/L) | 9.00 | 7.15-30.20 |
| PT (s) | 16.00 | 13.60-16.80 |
| APTT (s) | 53.60 | 37.00-68.55 |
| FIB (g/L) | 2.10 | 1.20-3.43 |
| INR | 1.30 | 1.02-1.35 |
| **Concomitant medications** |  |  |
| Meropenem | 7 |  |
| Omeprazole | 5 |  |
| Voriconazole | 1 |  |
| Fluconazole | 2 |  |
| Extracorporeal life support |  |  |
| CRRT | 1 |  |
| ECMO | 1 |  |

**Supplementary Table S3** The comparison results between the new model and the model by Yang et al.

| Item | New model | Yang et al. (2021) |
| --- | --- | --- |
| Number of subjects | 145 | 63 |
| Samples | 213 | 246 |
| Country (single/multiple) | China (single) | China (single) |
| Age (years) median [range] | 6.3 [0.2-15.2] | 3.85 [0.1-15.3] |
| Structural model | One-compartment model | Two-compartment model |
| PK parameters | CL (L/h) = 2.80 × (WT/20.00) ^0.69^ × (eGFR/126.39)^0.34^  V (L) = 14.48 × (WT/20.00) ^0.89^ | CL [L/h]=2.34 * (Weight/15)^0.8^ * (AST/45.9)^-0.16^  V_C_ [L]=5.22  Q [L/h]=7.14 * (Weight/15)^1.09^  V_P_ [L]=28.79 |
| Interindividual variability | CL=48.79% | CL=52.51%  V_C_=55.78%  Q =53.45% |
| Residual variability | Prop=40.12% | Prop=29% |
| Predictive performance |  |  |
| MDPE (%) | -14.41 | 18.17 |
| MAPE (%) | 37.48 | 56.91 |
| F_20_ (%) | 33.62 | 15.05 |
| F_30_ (%) | 49.10 | 22.58 |

PE%, the relative prediction error, PE% = (PRED-OBS)/OBS×100%; MDPE, the median of prediction error (median PE%); MAPE, the absolute median of prediction error (median |PE|%); F_20_, the percentage of PE% within ±20%; F_30_, the percentage of PE% within ±30%.

**Supplementary Table S4** The best-tuned hyperparameters for ML models

| Model | Hyperparameter | Value |
| --- | --- | --- |
| SVR |  |  |
|  | C | 4 |
|  | Epsilon | 0.06 |
|  | Gamma | 0.1 |
| GBDT |  |  |
|  | Learning_rate | 0.1 |
|  | Max_depth | 3 |
|  | N_estimators | 200 |
|  | Subsample | 0.7 |
|  | Min_samples_leaf | 2 |
|  | Min_samples_split | 5 |
| RF |  |  |
|  | Max_depth | 8 |
|  | N_estimators | 352 |
|  | Max_features | 0.7 |
|  | Min_samples_leaf | 7 |
|  | Min_samples_split | 10 |
| XGBoost |  |  |
|  | Learning_rate | 0.2 |
|  | Max_depth | 3 |
|  | N_estimators | 50 |
|  | Subsample | 0.7 |
| LightGBM |  |  |
|  | Learning_rate | 0.05 |
|  | N_estimators | 150 |
|  | Max_depth | 3 |
|  | Bagging_fraction | 0.6 |
|  | Num_leaves | 7 |
| AdaBoost | Learning_rate | 0.2 |
|  | N_estimators | 328 |
|  | Loss | Square |
| CatBoost |  |  |
|  | Depth | 4 |
|  | Iterations | 337 |
|  | L2_leaf_reg | 3 |
|  | Learning_rate | 0.1 |
|  | Border_count | 128 |


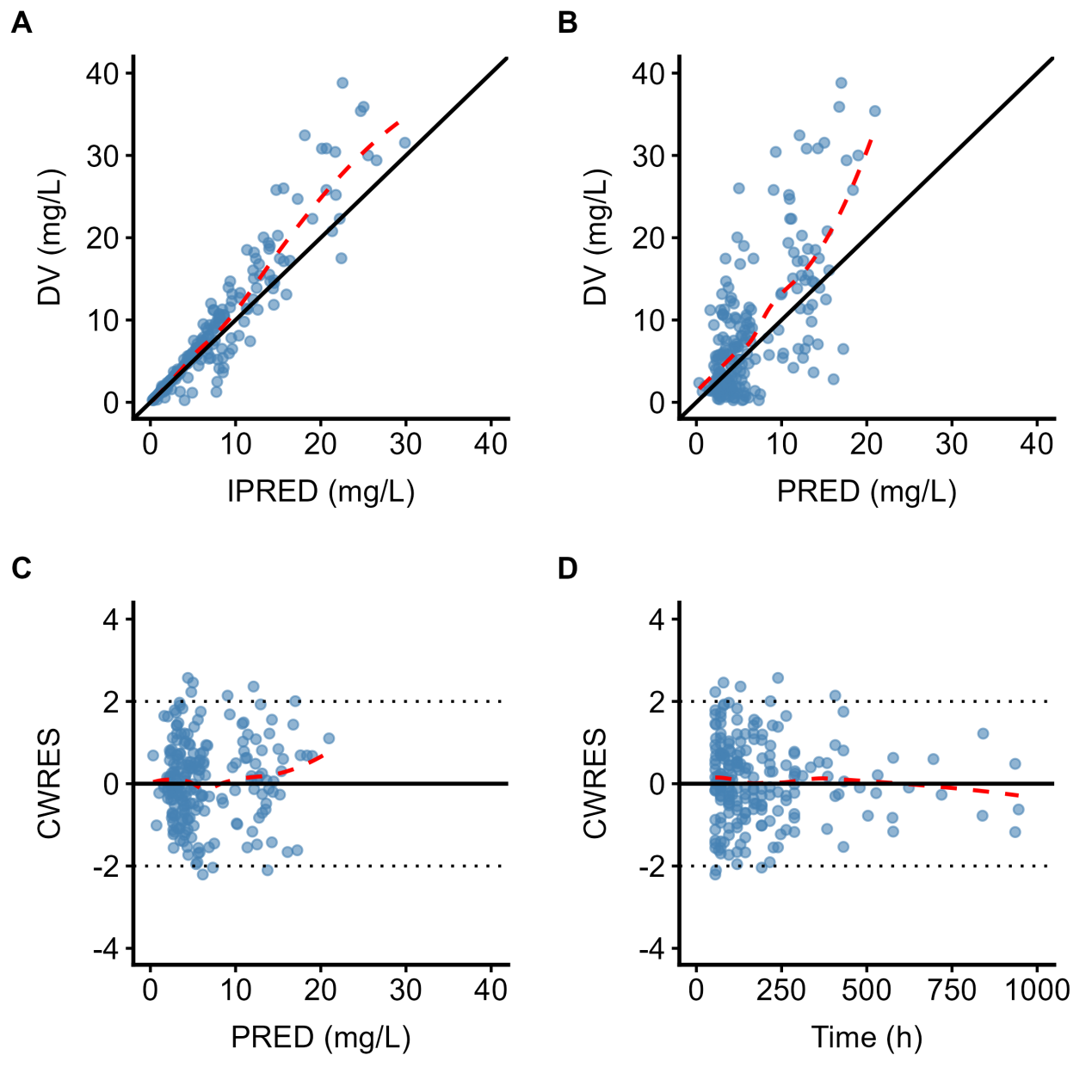


**Supplementary Figure S1** The goodness-of-fit plots of the final model. (A) Observed values versus individual predictions (DV vs. IPRED); (B) Observed values versus population predictions (DV vs. PRED); (C) Conditional weighted residuals versus population predictions (CWRES vs. PRED); (D) Conditional weighted residuals versus time (CWRES vs.Time).


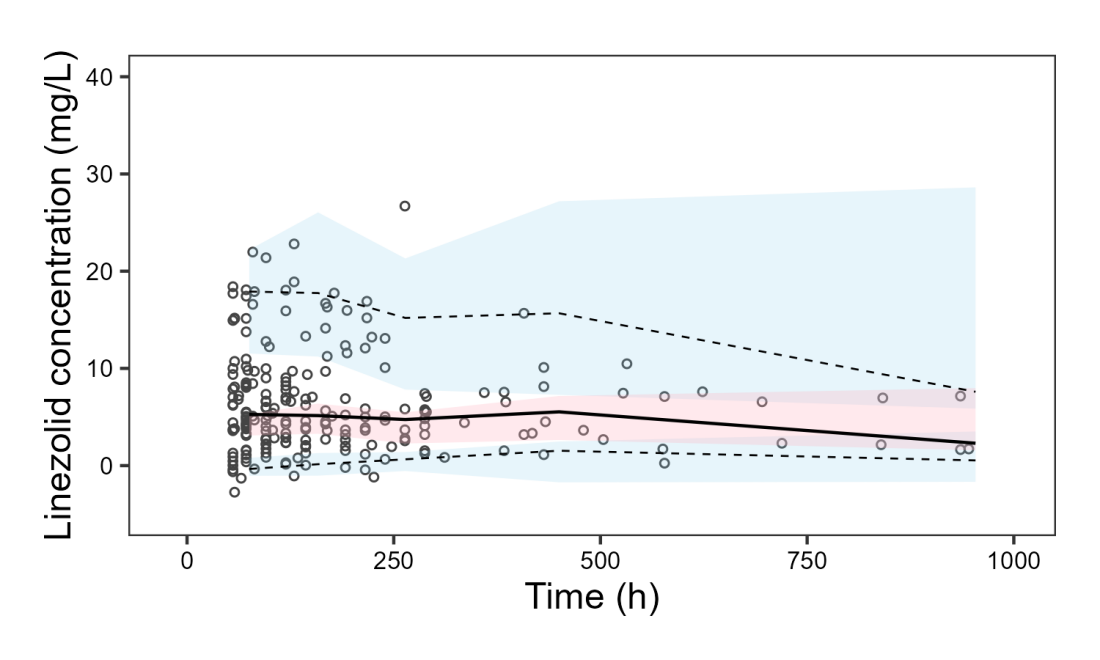


**Supplementary Figure S2** Prediction- and variability-corrected visual predictive check (pvcVPC, n=1000) of the final model. The black circles are the observed concentrations. The dashed black lines represent the 2.5 and 97.5th percentiles for the prediction-corrected concentrations. The solid black line represents the 50th percentile for the prediction-corrected concentrations. The shaded areas represent the 95% confidence interval.


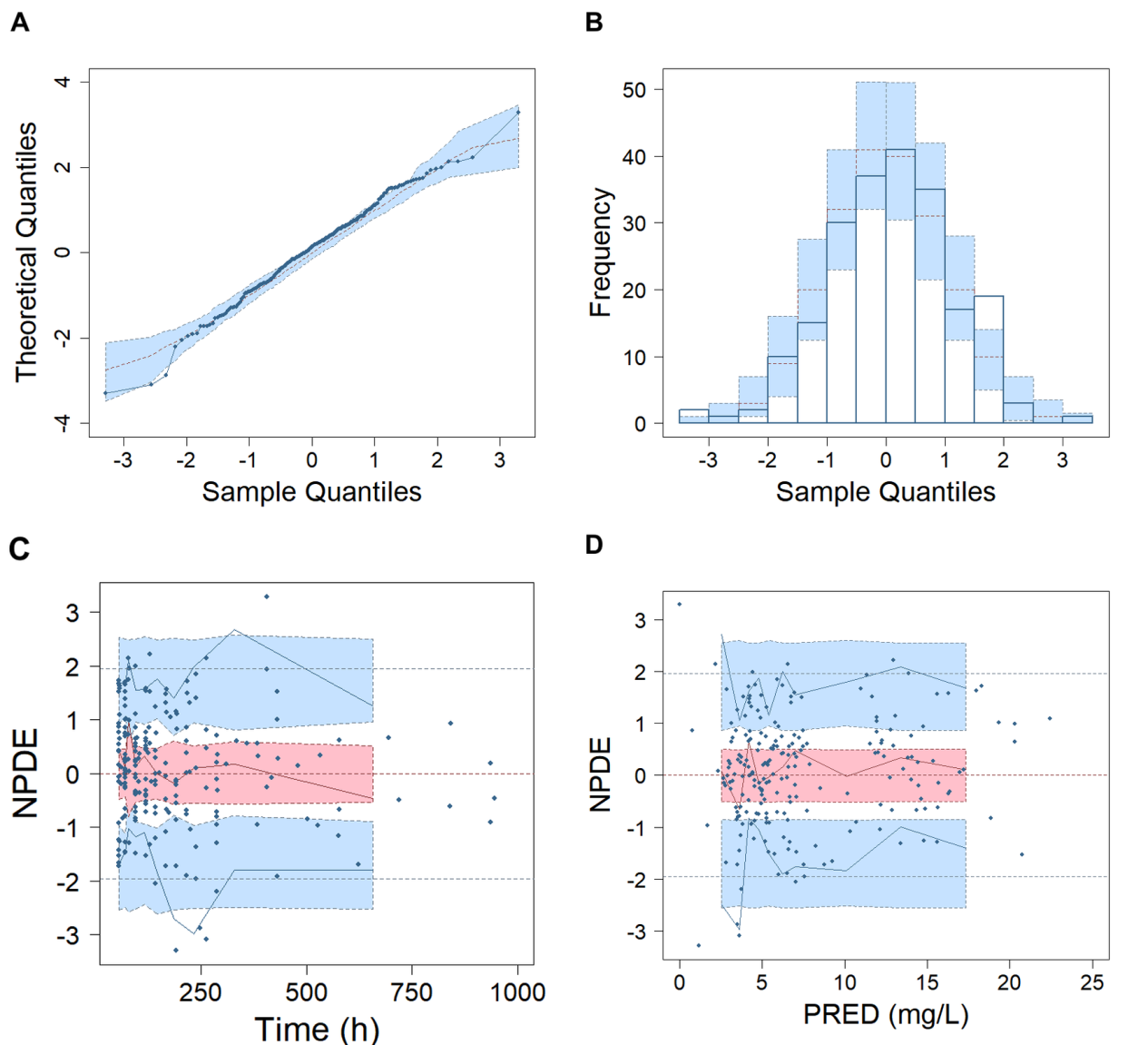


**Supplementary Figure S3** The results of normalized prediction distribution errors (NPDE) test. (A) Quantile-quantile plot of NPDE against the theoretical distribution; (B) Histogram of NPDE against the theoretical distribution; (C) Scatterplot of NPDE versus Time; (D) Scatterplot of NPDE versus PRED.


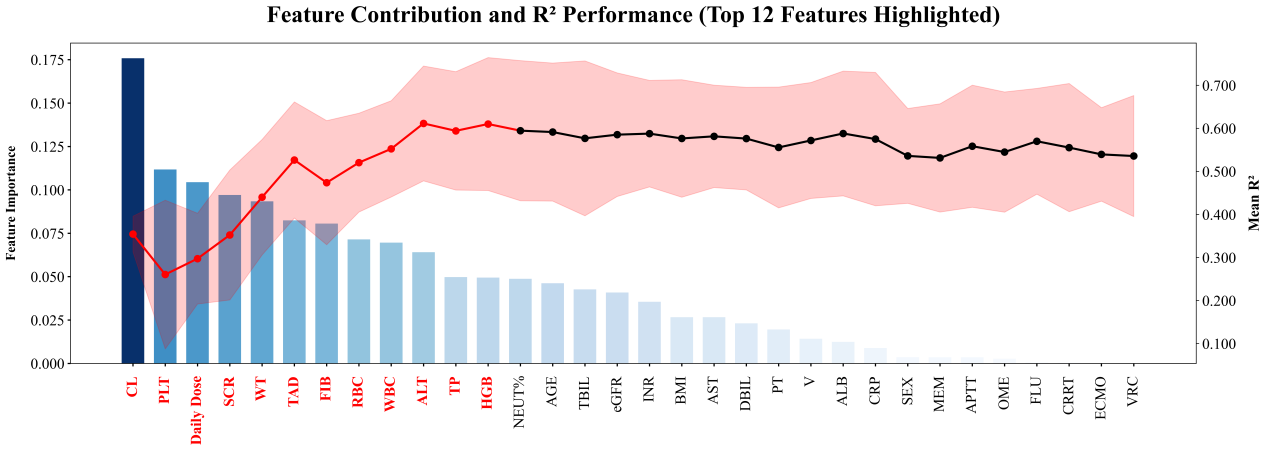


**Supplementary Figure S4** Feature selection schematic diagram. The optimal feature subset is identified through an integrated approach that combines an XGBoost regressor, forward feature selection, and cross-validation.


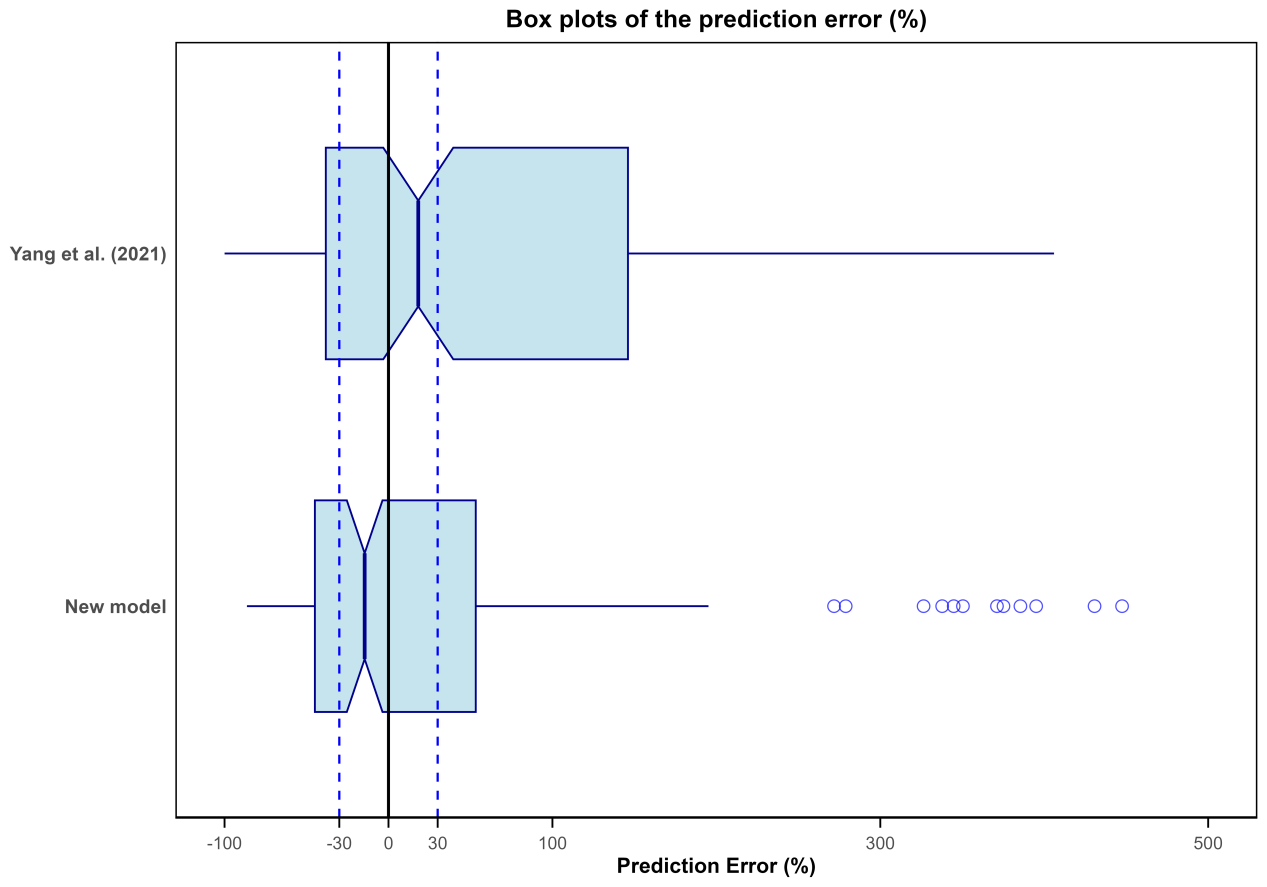


**Supplementary Figure S5** Box plots of prediction error (PE%) for the new model and the model by Yang et al. (2021). Black solid and blue dashed lines are reference lines indicating PE of 0% and±30%, respectively.
